# Repetitive Negative Thinking About Suicide: Associations With Lifetime Suicide Attempts

**DOI:** 10.32872/cpe.5579

**Published:** 2021-09-30

**Authors:** Tobias Teismann, Thomas Forkmann, Johannes Michalak, Julia Brailovskaia

**Affiliations:** 1 Mental Health Research and Treatment Center, Ruhr-Universität Bochum, Bochum, Germany; 2 Department of Clinical Psychology, University of Duisburg-Essen, Essen, Germany; 3 Department of Clinical Psychology and Psychotherapy, Universität Witten-Herdecke, Witten, Germany; Philipps-University of Marburg, Marburg, Germany

**Keywords:** repetitive negative thinking, rumination, suicide ideation, suicide attempts

## Abstract

**Background:**

Repetitive negative thinking has been identified as an important predictor of suicide ideation and suicidal behavior. Yet, only few studies have investigated the effect of suicide-specific rumination, i.e., repetitive thinking about death and/or suicide on suicide attempt history. On this background, the present study investigated, whether suicide-specific rumination differentiates between suicide attempters and suicide ideators, is predictive of suicide attempt history and mediates the association between suicide ideation and suicide attempts.

**Method:**

A total of 257 participants with a history of suicide ideation (55.6% female; Age M = 30.56, Age SD = 11.23, range: 18–73 years) completed online measures on suicidality, general and suicide-specific rumination.

**Results:**

Suicide-specific rumination differentiated suicide attempters from suicide ideators, predicted suicide attempt status (above age, gender, suicide ideation, general rumination) and fully mediated the association between suicide ideation and lifetime suicide attempts.

**Conclusion:**

Overall, though limited by the use of a non-clinical sample and a cross-sectional study design, the present results suggest that suicide-specific rumination might be a factor of central relevance in understanding transitions to suicidal behavior.

## Highlights

- Suicide-specific rumination was investigated in participants with a history of suicide ideation.
- Suicide-specific rumination differentiated suicide attempters from suicide ideators.
- Suicide-specific rumination predicted suicide attempt status.
- Suicide-specific rumination mediated the association between suicide ideation and lifetime suicide attempts.
- Suicide-specific rumination might be a factor of central relevance in understanding transitions to suicidal behavior.

Repetitive negative thinking (RNT) is defined as a style of thinking about one’s problems or negative experiences with three key characteristics: the thinking is repetitive, it is at least partly intrusive, and it is difficult to disengage from. Two additional features of RNT are that individuals perceive it as unproductive and it captures mental capacity (Ehring et al., 2011). The two most intensively studied types of RNT are worry and depressive rumination. RNT – in the form of rumination and worry – has been identified as a critical factor in the development and maintenance of psychiatric symptoms and disorders (Ehring & Watkins, 2008; Teismann & Ehring, 2019; Watkins, 2008). In prospective studies, rumination was found to predict the future onset of a major depressive episode (Nolen-Hoeksema, 2000; Nolen-Hoeksema et al., 2007; Robinson & Alloy, 2003; Wilkinson et al., 2013) and to mediate the effect of various risk factors on the onset of depression (Spasojević & Alloy, 2001). Additional studies have shown that rumination prospectively predicts the onset of post-traumatic stress disorder (Moulds et al., 2020; Szabo et al., 2017) and is linked to the maintenance of social anxiety disorder (Penney & Abbott, 2014), insomnia (Takano et al., 2014) and eating disorder psychopathology (Smith, Mason, & Lavender, 2018). Moreover, a close association between RNT, suicide ideation and suicide attempts has been shown in cross-sectional and longitudinal studies (Rogers & Joiner, 2017) – even when different types of RNT as well as different methodologies, samples (clinical and non-clinical) and measures of suicidality were used (Kerkhof & van Spijker, 2011; Law & Tucker, 2018). As such, rumination significantly predicted suicide ideation in prospective studies using student and community samples (Miranda & Nolen-Hoeksema, 2007; Smith, Alloy, & Abramson, 2006). Furthermore, rumination was found to be more common in suicide attempters than in non-attempters (e.g., Horwitz et al., 2019). Galynker (2017) understands intensive, persistent and uncontrollable brooding (*ruminative flooding*) as a core feature of an acute suicidal state, the so-called suicide crisis syndrome. Taken together, there is strong empirical evidence for the importance of RNT with respect to understanding suicide ideation and behavior.

In the vast majority of these studies, the relationship between general RNT and suicidal ideation and suicide attempts was investigated. However, Rogers and Joiner (2018a, 2018b) have recently started to study the effect of suicide-specific rumination, that is, RNT about death and/or suicide. They found that suicide-specific rumination is associated with lifetime suicide attempts over and above a large array of known risk factors, including suicide ideation, general rumination, depression and anxiety (Rogers & Joiner, 2018a). Furthermore, they could show that the association between suicide-specific rumination and lifetime suicide attempts is mediated by an acute suicidal state, called acute suicidal affective disturbance (ASAD; Rogers & Joiner, 2018b). In both of these studies, suicide-specific rumination was assessed using either a 5-item (Rogers & Joiner, 2018b) or an 8-item (Rogers & Joiner, 2018a) version of the Suicide Rumination Scale (SRS). This scale assesses the tendency to ruminate or fixate on one’s suicidal thoughts, intention and plans. However, it cannot be excluded that some items of the SRS may confound general preparation behavior (“When I have thoughts of suicide, I think about how I want to kill myself”; “… I wonder what the fastest and easiest way to die is”) or so called flash forwards (“When I have thoughts of suicide, I imagine the process of how I want to kill myself”), with generic features of RNT (“When I have thoughts of suicide, I have trouble getting the suicidal thoughts out of my mind”). It is therefore unclear whether the significant association between suicide-specific rumination – as assessed with the SRS – and lifetime suicide attempts are in fact due to RNT or rather a consequence of increased preparation and planning behavior.

On this background, the current study aims at investigating the association between suicide-specific rumination and suicidal behavior with a suicide-specific version of the Perseverative Thinking Questionnaire (PTQ; Ehring et al., 2011), a self-report measure designed to assess core characteristics of RNT (repetitiveness, intrusiveness, difficulties with disengagement, perceived unproductiveness). The study had three aims: 1. To investigate whether suicide-specific rumination – as assessed with an unconfounded measure – differentiates between lifetime suicide attempters and non-attempters; 2. To investigate, whether suicide specific rumination is associated with lifetime suicide attempts – above and beyond age, gender, current suicide ideation and general rumination; 3. To investigate whether suicide-specific rumination mediates the association between current suicide ideation and lifetime suicide attempts. Since most suicide ideators do not show suicidal behavior, the necessity to understand what differentiates attempters from ideators has recently been highlighted (May & Klonsky, 2016).

## Method and Materials

### Participants and Procedure

Between March and May 2019, *N* = 300 (58% female; *M*_age_= 32.25, *SD*_age_ = 13.68, range: 18–77 years) and again between February and June 2020, *N* = 276 (67% female; *M*_age_ = 32.08, *SD*_age_ = 10.73, range: 18–64 years) participants took part in a single assessment using an online survey. The assessments took part within the context of two other studies (Teismann & Brailovskaia, 2020; Teismann et al., 2020), that were advertised as investigating the association between well-being and psychological strain. It was assured that no participant took part in both of these studies. Of the two samples, *n* = 257 (55.6% female; *M*_age_ = 30.56, *SD*_age_ = 11.23, range: 18–73 years) reported lifetime suicide ideation and were included in the present study. One-hundred and twenty-nine participants (50.2%) reported some suicide ideation in the last four weeks (SSEV- score ≥1); fifty-two participants (20.2%) indicated that they had attempted suicide at least once in their lifetime (range: 1–6). All participants – except for one Asian participant – were Caucasian.

Participants were recruited through postings at local university as well as social media postings on Facebook and Twitter. Data was collected through an anonymous online survey using the SoSci-server (https://www.soscisurvey.de/). Participation in the study was not compensated; yet, participating students were eligible to receive course credits. In order to take part in the study, participants had to be at least 18 years old and to give their consent to participation at the beginning of the study. Prior to assessments, all participants were informed about the purpose of the study, the voluntary nature of their participation, data storage and security. The study was approved by the responsible Ethics Committee.

### Measures

#### Suicide Ideation and Behavior Scale (SSEV)

The SSEV (Teismann et al., 2021) assesses with six items the frequency of suicide ideation in the past four weeks (e.g., “During the past four weeks, … I thought it would be better if I wasn't alive, … I've been thinking about killing myself, … I have seriously considered killing myself”). All items are answered on a 6-point Likert scale ranging from “1=*never*” to “5=*many times every day*”, with higher scores indicating greater severity of suicidal ideation. Occurrence (“In the course of my life I have tried to kill myself - and I really wanted to die”) and number of lifetime suicide attempts (“How many times have you tried to kill yourself?”) are assessed with two further SSEV-items. The scale has been shown to have a good internal consistency (Cronbach’s α ≥ .92; Teismann et al., 2021). Accordingly, internal consistency was good in the current sample, (α = .84).

#### Perseverative Thinking Questionnaire (PTQ)

The PTQ (Ehring et al., 2011) is a 15-item self-report measure designed to assess process characteristics of perseverative thinking (“The same thoughts keep going through my mind again and again”; “I keep asking myself questions without finding an answer”; “Thoughts intrude into my mind”; “My thoughts take up all my attention”). All items are to be answered on a 5-point scale ranging from 0 (*“never”*) to 4 (*“almost always”*). The scale has been shown to have good internal consistencies (Cronbach`s α ≥ .93; Ehring et al., 2011). Accordingly, internal consistencies were excellent in the current sample, α = .95.

#### Perseverative Thinking about Suicide Questionnaire (PTSQ)

The PTSQ (Teismann, 2018) is modeled after the PTQ and assesses with nine items suicide specific rumination (“I can´t stop dwelling about suicide”; “I am thinking about suicide the whole time”; “Thoughts about suicide intrude into my mind”; “My thoughts about suicide repeat themselves”). In the adaption process the word “thoughts” from the original PTQ was replaced by the term “suicidal thoughts” in the PTSQ: For example the PTQ-item “The same thoughts keep going through my mind again and again” became the PTSQ-item “The same thoughts about suicide keep going through my mind again and again”. Items from the PTQ that were not adjustable in the described manner (i.e., “I think about many problems without solving any of them”) were not included in the PTSQ. The adaptation was conducted by the first author and consented with all co-authors. All items are to be answered on a 5-point scale ranging from 0 (*“never”*) to 4 (*“almost always”*). Participants are only asked to answer all these items, if they affirm a first screening item (“In my lifetime I have thought about suicide”). The scale has been shown to have high internal consistency (Cronbach`s α = .94; Höller et al., in preparation). Accordingly, an exploratory factor analysis (EFA) using principal component analysis (PCA; rotation method: varimax) revealed a unidimensional factor structure within the present sample as well as excellent internal consistency, α =.95.

### Statistical Analyses

Statistical analyses were conducted with SPSS 26 and the Process macro version 3.5 (www.processmacro.org/index.html; Rockwood & Hayes, 2020). Descriptive statistics and zero-order bivariate correlations between the investigated variables were calculated. Differences between groups (lifetime suicide ideators: *n* = 205 vs. lifetime attempters: *n* = 52) were analyzed using one-way ANOVAs. Considering the different sizes of both groups, Hedges’g was included as effect size (see Hedges, 1981). Notably, the current data fit the assumptions for the calculation of multivariate analyses (no significant outliners > 3 and < -3, number of significant outliners > 2 and < -2 below 5%; no violation of multicollinearity assumption as all values of tolerance > 0.25, and all variance inflation factor values < 5; interaction between the independent variables and their logarithmic transformations is not significant) (see Field, 2013; Tabachnick & Fidell, 2014; Urban & Mayerl, 2006). Next, a three-step multiple logistic regression analysis was calculated to examine the relative contribution of current suicide ideation (SSEV), general rumination (PTQ) and suicide-specific rumination (PTSQ) to the prediction of lifetime suicide attempt status (coded: 0 = no attempts, 1 = attempts). The variable age was significantly correlated with current suicide ideation (*r* = -.163, *p* < .01), general rumination (*r* = -.189, *p* < .05), and suicide-specific rumination (*r* = -.161, *p* < .05). The variable gender (coded: 0 = woman, 1 = man) was negatively correlated with general rumination (*r* = -.147, *p* < .05), and lifetime suicide attempt status (*r* = -.129, *p* < .05). Considering the relationships of age and gender with the potential predictors and the outcome of the regression model, both were included as control variables. Thus, age and gender were included in Step 1 of the regression model, current suicide ideation and general rumination were included in Step 2, and suicide-specific rumination was included in Step 3. Finally, a mediation analysis was conducted that included current suicide ideation (predictor), suicide-specific rumination (mediator), and number of lifetime suicide attempts (outcome). The basic association between current suicide ideation and lifetime suicide attempts was denoted by *c* (the total effect). The path of current suicide ideation to suicide specific rumination was denoted by *a*, and the path of suicide specific rumination to lifetime suicide attempts was denoted by *b*. The combined effect of path *a* and path *b* presented the indirect effect. The direct effect of current suicide ideation on lifetime suicide attempts after inclusion of suicide specific rumination in the model was denoted by *c’*. The mediation effect was assessed by the bootstrapping procedure (10.000 samples) that provides percentile bootstrap confidence intervals (95% CI).

## Results

### Descriptive Statistics, Correlations and Group Differences

Descriptive statistics for each measure and correlations are presented in Table 1. Correlation analyses indicated that all study variables correlated significantly with each other (see Table 1). The correlations ranged between *r* = .354 and *r* = .806 (all: *p* < .01), indicating medium to large effects (see Cohen, 1988).

**Table 1:** Means, Standard Deviations and Correlations of Study Variables

Measure	*M* (*SD*)	*Min–Max*	Skewness	Kurtosis	2	3	4
**1. SSEV**	8.00 (3.35)	6–23	2.130	4.449	.354**	.806**	.406**
**2. SSEV-SA**	0.32 (0.84)	0–6	3.932	18.382	–	.463**	.265**
**3. PTSQ**	15.07 (7.32)	9–44	1.509	1.813		–	.490**
**4. PTQ**	47.04 (13.07)	16–75	-.043	-.448			–

*Note*. *N* = 257; *M* = Mean; *SD* = Standard Deviation; *Min* = Minimum; *Max* = Maximum; SSEV = Suicide Ideation and Behavior Scale; SSEV-SA = Suicide Ideation and Behavior Scale – lifetime number of suicide attempts; PTSQ = Perseverative Thinking about Suicide Questionnaire; PTQ = Perseverative Thinking Questionnaire. SSEV-SA was dichotomized (0 = no attempts, 1 = attempts) for the correlation analyses.

**p < .01.

Lifetime suicide ideators (assessed with the PTSQ-screening item) and lifetime suicide attempters differed significantly in PTSQ-scores (suicide ideators: *n* = 205; *M* = 13.50, *SD* = 5.88, range: 9–36; suicide attempters: *n* = 52; *M* = 21.29, *SD* = 9.02, range: 9–44), *F*(1,255) = 57.52, *p* < .001, effect size: Hedges’*g* = 1.17 (large effect). Furthermore, lifetime suicide ideators (*M* = 45.36, *SD* = 12.61, range: 16–75) and lifetime suicide attempters (*M* = 53.67, *SD* = 12.89, range: 23–75) differed significantly in PTQ-scores, *F*(1,255) = 17.89, *p* < .001, effect size: Hedges’*g* = 0.65 (medium effect); with suicide attempters reporting more RNT than suicide ideators.

### Prediction of Lifetime Suicide Attempts

Associations between study variables and lifetime suicide attempts are shown in Table 2. In the multiple logistic regression model, current suicide ideation (*OR*: 1.19; small effect, see Chen, Cohen, & Chen, 2010) and general rumination (*OR*: 1.03; small effect, see Chen et al., 2010) served as a significant predictor of lifetime suicide attempts in Step 2. However, in Step 3, only the new included variable suicide-specific rumination emerged as a significant predictor of lifetime suicide attempts (*OR*: 1.14; small effect, see Chen et al., 2010).

**Table 2:** Results From a Three-Step Multiple Logistic Regression Analysis Predicting Lifetime Suicide Attempts (Dichotomized: 0 = no attempts, 1 = attempts)

Step	*OR* (95% CI)	*p*
Step 1		
Age	0.98 [0.95-1.01]	.163
Gender	0.47 [0.25-0.90]	.023
Step 2		
Age	0.99 [0.96-1.03]	.720
Gender	0.56 [0.28-1.14]	.110
SSEV	1.19 [1.09-1.31]	< .001
PTSQ	1.03 [1.00-1.06]	.047
Step 3		
Age	1.00 [0.96-1.03]	.796
Gender	0.50 [0.24-1.04]	.065
SSEV	0.97 [0.85-1.12]	.719
PTQ	1.01 [0.98-1.04]	.461
PTSQ	1.14 [1.06-1.23]	< .001

*Note*. *N* = 257; SSEV-SI = Suicide Ideation and Behavior Scale; PTQ = Perseverative Thinking Questionnaire; PTSQ = Perseverative Thinking about Suicide Questionnaire; *OR* = odds ratio from logistic regression; *CI* = confidence interval.

### Mediation Analysis

**Figure 1:**
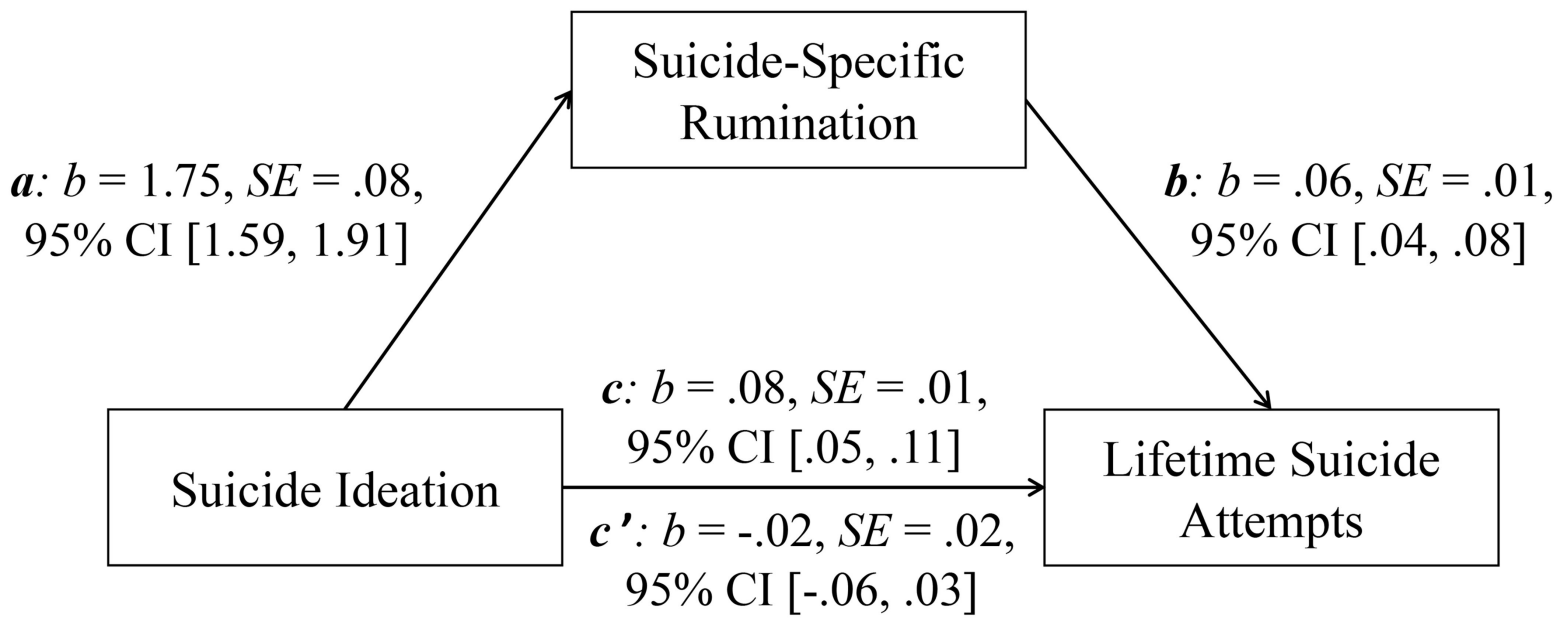
Mediation Model With Suicide Ideation (Predictor), Suicide-Specific Rumination (Mediator), and Lifetime Suicide Attempts (Outcome). *Note. c* = total effect; *c’* = direct effect; *b* = standardized regression coefficient; *SE* = standard error; CI = confidence interval.

Figure 1 shows results of the bootstrapped mediation analysis. The basic relationship between current suicide ideation (predictor) and lifetime suicide attempts (outcome) was significant (total effect, *c: p* < .001). The association between current suicide ideation and suicide-specific rumination (mediator) (*a: p* < .001), as well as the link between suicide-specific rumination and lifetime suicide attempts (*b: p* < .001) were also significant. In contrast, the relationship between current suicide ideation and lifetime suicide attempts was no longer significant after the inclusion of suicide-specific rumination in the model (direct effect, *c’: p* = .445). The indirect effect (*ab*) was significant, *b* = .10, *SE* = .03, 95% CI [.04, .17]. Thus, suicide-specific rumination significantly mediated the relationship between current suicide ideation and lifetime suicide attempts.

## Discussion

The present study investigated the association between RNT – that is, suicide-specific rumination and general rumination – and (lifetime) suicide attempts. The main findings were as follows: (1.) General rumination and suicide-specific rumination differentiated between lifetime suicide attempters and suicide ideators; (2.) Suicide-specific rumination was predictive of lifetime suicide attempt status – controlling for age, gender, current suicide ideation and general rumination; (3.) The association between current suicide ideation and lifetime suicide attempts was fully mediated by suicide-specific rumination.

These results complement previous research showing an association between general rumination and suicide ideation/behavior (Rogers & Joiner, 2017) as well as between suicide-specific rumination and lifetime suicide attempts (Rogers & Joiner, 2018a, 2018b). In accordance with findings by Rogers and Joiner (2018a) it was shown that suicide-specific rumination outperformed other suicide risk factors – including current suicide ideation – in the prediction of lifetime suicide attempt status. Of note, findings could be replicated with a new – potentially unconfounded – measure of suicide-specific rumination. Though further study results have to be awaited, these findings suggest a rather robust effect of suicide-specific rumination. Accordingly, it seems as if RNT about suicide may be more pernicious in increasing the risk for suicidal behavior than ruminative thoughts about one’s distress more generally.

Nonetheless, both general rumination and suicide-specific rumination differentiated between (lifetime) suicide attempters and (lifetime) suicide ideators (cf., Horwitz et al., 2019). Klonsky and May (2015) recently emphasized that it is crucial to understand factors that differentiate those who consider suicide from those who make suicide attempts. Yet, in a comprehensive meta-analysis May and Klonsky (2016) found only few studies that directly compared suicide ideators and suicide attempters and only few variables that differentiated the two groups. Though the importance of single factors in differentiating suicide attempters and suicide ideators has recently been disputed (Huang et al., 2020), these findings point to the potential potency of (suicide-specific) RNT in understanding transitions to suicidal behavior.

A further analysis showed that the association between current suicide ideation and (lifetime) suicide attempts is completely mediated by suicide-specific rumination, that is, the risk of suicidal behaviour only increases when suicide is considered in a repetitive way. Within the metacognitive theory of emotional disorders, Wells and Matthews (2015) state that a psychological disorder results from an unhelpful thinking style called the Cognitive Attentional Syndrome (CAS). The CAS incorporates worry/rumination, threat monitoring and unhelpful thought control strategies. According to the theory, not single thoughts, assumptions or beliefs create emotional turmoil, but the way a person deals with these thoughts: only if respective thoughts activate the CAS, emotional and behavioral problems will follow. On this background one may assume that thoughts of suicide per se do not pose a great risk for suicidal behaviour (cf., McHugh et al., 2019), unless individuals engage in such thoughts in a repetitive manner. In future studies, the association between suicide-specific rumination and other variables of the metacognitive model should be investigated more closely.

The results of the current study should be interpreted with consideration of the following limitations. First, the PTSQ was developed for the current study and has only recently been subjected to stringent psychometric evaluation (Höller et al., in preparation). However, no direct comparison between the PTSQ and the Suicide Rumination Scale (SRS; Rogers & Joiner, 2018a) was made. Therefore, no conclusions with respect to the relationship between the two measures can be drawn, or determined whether one of the two measures is more valid in assessing suicide-specific rumination. Second, Rogers and Joiner (2018a) included a large number of control variables (e.g., depression, anxiety, insomnia, agitation, emotion regulation, general RNT) in their study on suicide specific RNT, whereas in the present study only age, gender, general RNT and current suicide ideation were included as control variables. Future studies should therefore strive to investigate, whether suicide specific RNT – as assessed with the PTSQ – also outperforms such a great number of suicide risk factors in predicting the presence of a lifetime suicide attempt. Third, general RNT is understood as a trait (Watkins & Nolen-Hoeksema, 2014) and both the PTSQ and the SRS capture suicide specific RNT in a trait-like manner. Nevertheless, it is unclear whether suicide specific RNT is indeed stable over time and across suicidal crises and/or whether it is (only) associated with more intense suicidal crises (cf., Galynker, 2017). Prospective studies with repeated measurements are needed. Fourth, all of the constructs included in this study were measured exclusively via self- report assessments. Although it may be difficult to gather information regarding the frequency of particular thought patterns, participants may be prone to inaccuracy and uncertainty when responding to self-report items. Finally, the use of a cross-sectional research design and a sample comprised of predominantly Caucasians, limits the generalizability of the results and the discussion of temporal/causal relationships between study variables. This limitation is of specific importance considering the interpretation of the results of the mediation analysis: As all data were collected at a single measurement time-point and the outcome measure (i.e., lifetime suicide attempts) is retrospective, it might be more appropriate to frame the findings as indirect effects rather than as mediation effects. A replication of this study in treatment-seeking samples with prospective research designs would help to indicate whether the study results remain consistent in more at-risk populations. Still, it is important to emphasize that all participants within the current study reported lifetime suicidal ideation, and in this sense are a group of clinical interest.

Not least therefore, the current study does exhibit potential clinical implications: First of all, it might be important to account for the presence of suicide-specific rumination in addition to other risk factors, when assessing individuals for suicide risk. Furthermore, suicide-specific rumination may be a potential target in treatment to reduce one’s suicidality. As such, (general) rumination has been shown to be malleable through treatments such as cognitive behavioral therapy (Teismann & Ehring, 2019) or mindfulness-based cognitive therapy (Gu et al., 2015). Therefore, it should be tested, whether suicide-specific rumination might be modifiable by similar interventions and techniques than general rumination. On the background of findings regarding the relevance of (suicide-specific) RNT in understanding suicidal behavior, respective studies seem highly warranted. Should the current findings be confirmed in further studies, it also seems reasonable to integrate suicide-specific rumination as a relevant factor with respect to the transition from suicide ideation to suicidal behavior within the current models of suicide ideation/behavior (cf., O’Connor & Kirtley, 2018).

## Supplementary Materials

Perseverative Thinking about Suicide Questionnaire (PTSQ). The PTSQ is modeled after the Perseverative Thinking Questionnaire (Ehring et al., 2011) and assesses with nine items suicide specific rumination (for access see Index of Supplementary Materials below).

10.23668/psycharchives.5036 Supplement 1 Supplementary materials to "Repetitive negative thinking about suicide: Associations with lifetime suicide attempts" [Questionnaire]

TeismannT.
ForkmannT.
MichalakJ.
BrailovskaiaJ.
 (2021). Supplementary Materials to "Repetitive negative thinking about suicide: Associations with lifetime suicide attempts"
[Questionnaire]. PsychOpen. 10.23668/psycharchives.5036
PMC966722936398103
