# Supplementary material for: Repetitive Negative Thinking About Suicide: Associations With Lifetime Suicide Attempts
Source: Clin Psychol Eur. 2021 Sep 30;3(3):e5579. doi: 10.32872/cpe.5579 (PMC9667229; doi:10.32872/cpe.5579)
Supplement: Supplement 1 [file cpe-03-5579-s01.pdf]

Perseverative **T**hinking about **S**uicide **Q**uestionnaire **(PTSQ*)**

Associate publication: Teismann, T., Forkmann, T., Michalak, J. & Brailovskaia, J. (2021). Repetitive negative thinking about suicide: Associations with lifetime suicide attempts. *Clinical Psychology in Europe*. https://doi.org/10.32872/cpe.5579

In diesem Fragebogen geht es darum, wie Sie lebensmüde Gedanken und Suizidgedanken typischerweise erleben. Bitte lesen Sie folgende Aussagen und geben Sie an, in welchem Ausmaß diese auf Sie zutreffen.
[In this questionnaire, you will be asked to describe how you *typically* think about suicide. Please read the following statements and rate the extent to which they apply to you when you think about suicide.]

Haben Sie schon einmal an Suizid gedacht oder sich ausgemalt durch einen Suizid zu sterben?
[Have you ever had thoughts of suicide or pictured yourself dying by suicide?]

☐ Ja [Yes] ☐ Nein [No]

Wenn ja, dann fahren Sie fort. Wenn nein, dann beenden Sie die Befragung.
[If yes, continue. If no, skip to end.]

		**nie** [never]	**selten** [rarely]	**manchmal** [sometimes]	**häufig** [often]	**fast immer** [almost always]
1.	Suizidgedanken gehen mir immer und immer wieder durch den Kopf. [The same thoughts about suicide keep going through my mind again and again.]	0	1	2	3	4
2.	Suizidgedanken drängen sich mir auf. [Thoughts about suicide intrude into my mind.]	0	1	2	3	4
3.	Ich kann nicht aufhören, über Suizid nachzudenken. [I can`t stop dwelling about suicide.]	0	1	2	3	4
4.	Meine Suizidgedanken wiederholen sich. [My thoughts about suicide repeat themselves.]	0	1	2	3	4
5.	Suizidgedanken tauchen auf, ohne dass ich dies will. [Thoughts about suicide come to my mind without me wanting them to.]	0	1	2	3	4
6.	Ich hänge an Suizidgedanken fest und kann mich nicht davon lösen. [I get stuck on thoughts about suicide and can`t move on.]	0	1	2	3	4
7.	Ich denke die ganze Zeit über Suizid nach. [I keep thinking about suicide all the time.]	0	1	2	3	4
8.	Suizidgedanken treten ganz plötzlich auf. [Thoughts about suicide just pop into my mind.]	0	1	2	3	4
9.	Ich fühle mich gezwungen, immer weiter über Suizid nachzudenken. [I feel driven to continue dwelling about suicide.]	0	1	2	3	4

* The PTSQ is modeled after the Perseverative Thinking Questionnaire (Ehring, T., Zetsche, U., Weidacker, K., Wahl, K., Schönfeld, S., & Ehlers, A. (2011). The Perseverative Thinking Questionnaire (PTQ): validation of a content-independent measure of repetitive negative thinking. *Journal of Behavior Therapy and Experimental Psychiatry*, *42*, 225–232.
